# Renal denervation beyond resistant hypertension: should drug intolerance redefine eligibility?

**DOI:** 10.1016/j.ijcrp.2026.200717

**Published:** 2026-10-06

**Authors:** Elena-Mihaela Cordeanu, Emma Morisot, Corina Mirea, Eric Prinz, Francois Bronner, Dominique Stephan

**Affiliations:** a Department of Hypertension and Vascular Diseases, Clinical Pharmacology, Strasbourg University Hospitals, Strasbourg, France; b Translational Cardiovascular Medicine, UR 3074, CRBS, University of Strasbourg, Strasbourg, France; c Investigation Network on Venous Thrombo-Embolism-French Clinical Research Infrastructure Network F-CRIN INNOVTE, Saint-Étienne, France; d Department of Nephrology, Strasbourg University Hospitals, Nouvel Hôpital Civil, Strasbourg, France; e Department of Cardiology, Strasbourg University Hospitals, Strasbourg, France

**Keywords:** Renal denervation, Drug intolerance, Uncontrolled hypertension, Sympathetic activation, Shared decision-making

## Abstract

Catheter-based renal denervation (RDN) is endorsed by major international guidelines as an adjunct to antihypertensive therapy, yet most regulatory and reimbursement frameworks still restrict its use to true resistant hypertension, usually defined as uncontrolled blood pressure (BP) despite three or more maximally tolerated antihypertensive drugs including a diuretic. This restriction leaves a clinically important population without an effective escalation pathway: patients with uncontrolled, high-risk hypertension who cannot tolerate the multi-class drug regimens required to qualify as resistant. We argue that this exclusion is increasingly difficult to justify. Contemporary sham-controlled trials enrolling patients on no or limited antihypertensive therapy demonstrate modest but reproducible ambulatory BP reductions in precisely the pharmacological context in which drug-intolerant patients are managed and guidelines published since 2023 acknowledge uncontrolled hypertension on fewer drugs or drug intolerance as clinically relevant scenarios for RDN. The device-attributable ambulatory systolic effect converges on 4-6 mmHg, 25–40% of treated patients derive no meaningful BP reduction, no pre-procedural variable reliably identifies responders, and no trial has been powered for cardiovascular outcomes. Drug intolerance also lacks an objective definition and frequently reflects undisclosed non-adherence. We propose a pragmatic referral pathway in which biochemical adherence testing and a documented trial of a low dose single-pill combination are mandatory steps, followed by out-of-office BP confirmation, secondary hypertension screening, anatomical eligibility and multidisciplinary shared decision-making.

## 1 The unmet need of drug-intolerant uncontrolled hypertension

Despite a broad and effective pharmacological armamentarium, only about half of treated hypertensive patients reach blood pressure (BP) targets [1,2]. Among the causes, drug intolerance is consistently underestimated and rarely incorporated into trial inclusion criteria or reimbursement frameworks [3]. Discontinuation rates for individual antihypertensive classes range from 4% to 28% depending on agent and population, with pooled estimates suggesting that multiple drug intolerance syndrome affects approximately 10% of patients with arterial hypertension [[3], [4], [5]]. These difficult-to-treat patients are not resistant in the canonical sense, as they cannot be uptitrated to three or more drug classes, yet their BP remains uncontrolled and their cardiovascular risk continues to accumulate.

Catheter-based RDN is a durable, adherence-independent intervention supported by randomized sham-controlled evidence [6,7]. Its endorsement by the European Society of Hypertension (ESH) 2023 guidelines [8], the European Society of Cardiology (ESC) 2024 guidelines [9], and the AHA/ACC 2025 guideline [10] is clear, yet access remains tied to resistant hypertension in many countries. This creates a paradox: the patient who cannot tolerate the drugs required to meet the formal definition of resistant hypertension may be excluded from RDN indications, precisely because of the intolerance that makes a non-pharmacological adjunctive therapy most relevant.

The controversy addressed in this article is therefore not whether RDN should replace pharmacological therapy. The question is whether documented intolerance to two or more first-line antihypertensive classes should itself trigger referral for RDN assessment when BP remains uncontrolled, cardiovascular risk is increased and secondary causes have been excluded. We argue that such patients sit at the intersection of an evolving guideline framework and a still conservative reimbursement structure, and that their evaluation should be based on phenotype, risk and shared decision-making rather than drug count alone.

The historical controversy around RDN efficacy must also be interpreted correctly. The negative SYMPLICITY HTN-3 trial was a methodological turning point, but it did not invalidate the renal sympathetic ablation concept. It exposed weaknesses of first-generation RDN research: reliance on office BP, susceptibility to white-coat effect and regression to the mean, medication inconsistencies, variable operator experience and incomplete energy delivery [6,11,12]. Contemporary second-generation RDN trials not only improved devices, but also BP phenotyping. By prioritizing ambulatory BP monitoring (ABPM), sham control, standardized procedural protocols and better patient selection, they reduced the noise that made early office BP-driven results difficult to interpret [6,[13], [14], [15], [16], [17], [18], [19], [20]]. Furthermore, procedural heterogeneity in early studies likely contributed to under-treatment: circumferential ablation was often incomplete and distal branch treatment was not systematic. Renal reinnervation is documented in animal models and remains biologically plausible in humans; its clinical significance is uncertain. Observational follow-up has reported sustained BP lowering up to ten years, but these ultra-long-term datasets are small (n = 39 completers in the largest single-center series), uncontrolled and vulnerable to survivor and attrition bias and should therefore be read as hypothesis-generating rather than proof of durability [21,22]. If BP effect is lost over time, whether through partial reinnervation or disease progression, the drug-intolerant patient has little pharmacological reserve. Yet, repeat RDN has been reported only in small case series, with no established protocol, no randomized efficacy or safety data, and no validated means of distinguishing reinnervation from disease progression.

### 1.1 Why RDN may be relevant in drug-intolerant hypertension

Renal sympathetic overactivity drives hypertension through two reinforcing mechanisms. Efferent fibers stimulate juxtaglomerular β1 receptors, triggering renin release, tubular sodium retention, renal vasoconstriction and a rightward shift of the pressure-natriuresis curve. Afferent fibers, activated by renal ischemia, hypoxia and inflammation, reinforce central sympathetic outflow via the rostral ventrolateral medulla. The resulting whole-body adrenergic drive accelerates vascular remodeling and hypertension-mediated organ damage [6,8,23].

Pharmacological strategies act at single points along this axis: renin-angiotensin system blockers neutralize the efferent humoral arm but require daily intake at therapeutic doses; beta-blockers attenuate central sympathetic effects but at the cost of metabolic, sexual and fatigue-related side effects that are particularly poorly tolerated in younger active patients (Fig. 1) [8,24]. In theory, patients can always be switched to other agents. In practice, multi-class intolerance often spans the main pharmacological pillars and pushes clinicians toward later-line drugs with weaker evidence or poorer tolerability [5,8,23].

**Fig. 1:**
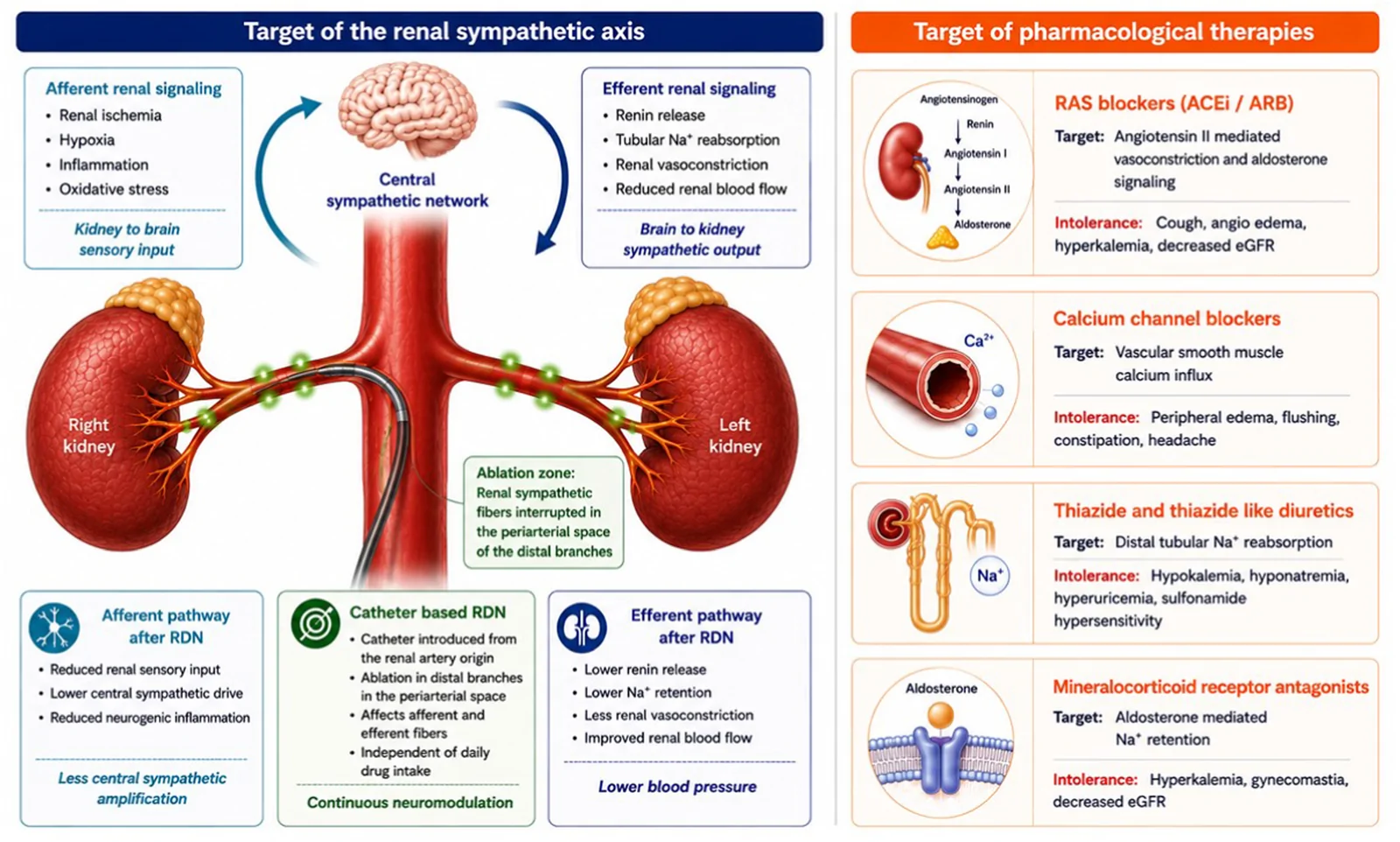
Mechanisms of action of catheter-based renal denervation versus pharmacological therapy. RDN interrupts both afferent and efferent renal sympathetic fibers in the periarterial space, providing a sustained, adherence-independent reduction in sympathetic tone (left panel). The four main antihypertensive classes act at single downstream points, each with a class-specific intolerance profile (right panel). ACEi: angiotensin-converting enzyme inhibitor; ARB: angiotensin receptor blocker; eGFR: estimated glomerular filtration rate; RAS: renin-angiotensin system; RDN: renal denervation.

RDN interrupts both afferent and efferent sympathetic fibers in the adventitia of the renal arteries, producing a sustained reduction in sympathetic tone that is, by design, “always on” [6,7,23]. This property is biologically valuable in the intolerant patient for three reasons. First, the intolerant patient is by definition exposed to suboptimal pharmacological coverage of the same neuro-hormonal cascade RDN modulates, thus RDN acts on a pathway that is, in these patients, incompletely addressed pharmacologically. This provides a biological rationale, not a demonstration of clinical benefit. Second, the BP-lowering effect of RDN is independent of medication adherence [7], which is consistently compromised in patients managing side effects [3,11,25]. Third, the reported durability of the response, maintained at three years in the SPYRAL HTN-ON MED and SYMPLICITY HTN-3 cohorts [26,27] and out to ten years in observational cohorts [21], addresses the long-term tolerability and efficacy. However, in SPYRAL HTN-ON MED the between-group difference was not significant at 12 months, the last blinded timepoint, and widened only after unblinding and crossover of part of the sham arm [27].

### 1.2 Trial evidence in lightly treated patients

The effect of RDN in patients on no or limited antihypertensive medication appears comparable to its effect in resistant hypertensive patients. The most rigorous test of RDN efficacy in patients who are not on full triple therapy comes from off-medication and on-medication sham-controlled trials of second-generation devices. These studies were not designed as dedicated drug-intolerance trials. However, they are directly relevant because they test RDN in the same pharmacological space in which intolerant patients live: uncontrolled BP despite absent, reduced or limited medication exposure (Table 1).

**Table 1:** Sham-controlled randomized trials of second-generation RDN devices specifically relevant to the lightly-treated/intolerant patient.

Trial	Device/energy	Population (background)	N (RDN/sham)	Change in ABPM SBP from baseline, RDN vs. sham (mmHg)
SPYRAL HTN-OFF MED Pivotal [16]	Symplicity Spyral (RF)	Mild-moderate HTN, off all antihypertensives	166/165	−4.7 vs. −0.6 (24h SBP, 3 mo) p = 0.0005
RADIANCE-HTN SOLO [15]	Paradise (US)	Uncontrolled HTN, 0-2 drugs (after washout)	74 /72	−8.5 vs. −2.2 (daytime, 2 mo) p < 0.001
RADIANCE II [14]	Paradise (US)	Uncontrolled HTN, ≤2 drugs (after washout)	150 /74	−7.9 vs. −1.8 (daytime, 2 mo) p < 0.0001
SPYRAL HTN-ON MED (pilot) [17]	Symplicity Spyral (RF)	Uncontrolled HTN, 1-3 drugs, adherence verified	38 /42	−9.0 vs. −1.6 (24h SBP, 6 mo) p = 0.006
SPYRAL HTN-ON MED Expansion [18]	Symplicity Spyral (RF)	Uncontrolled HTN, 1-3 drugs	168/89	−5.9 vs. −5.8 (24h SBP, 6 mo) p = 0.97
TARGET BP I [28]	Peregrine (alcohol)	Uncontrolled HTN, 2-5 drugs including a diuretic	148 /153	−10.0 vs. −6.8 (24 h SBP, 3 mo) difference −3.2 mmHg; p = 0.0487

ABPM: ambulatory BP monitoring; HTN: hypertension; mo: months; N: number; RDN: renal denervation; RF: radiofrequency; SBP: systolic BP; US: ultrasound.

Table footnote: Values shown are within-arm changes from baseline. Between-group differences quoted in the text are adjusted for baseline BP and therefore differ slightly from the arithmetic difference between the two columns. Reported p-values refer to the between-group comparison.

SPYRAL HTN-OFF MED Pivotal (n = 331) randomized patients with mild-to-moderate hypertension off any antihypertensive therapy and demonstrated a baseline-adjusted treatment difference of −3.9 (−6.2 to −1.6) mmHg in 24-h ambulatory systolic BP at three months and of −6.5 mmHg in office systolic BP (−9.6 to −3.5), with a posterior probability of superiority above 0.999 [16]. SPYRAL HTN-ON MED, in patients on 1-3 antihypertensives with verified adherence by detectable urinary drug metabolites, showed a 7.0 mmHg between-group difference in 24-h ambulatory systolic BP at six months [17]; in the same cohort, ambulatory systolic and diastolic BP remained lower than sham at 24 and 36 months despite similar antihypertensive treatment intensity [27], with a parallel reduction in morning and nighttime BP [29]. The SPYRAL HTN-ON MED Expansion cohort, however, did not reproduce a significant 24-h ambulatory effect at six months (−5.9 vs. −5.8 mmHg; p = 0.97) [18]. Medication burden was higher in the sham arm at six months (3.5 vs. 2.9; p = 0.043), and biochemical drug testing detected an increase in 26% of sham compared with 17% of RDN patients, which the investigators considered sufficient to mask the ambulatory treatment effect. RADIANCE-HTN SOLO (n = 146) and the larger pivotal RADIANCE II (n = 224), both employing ultrasound RDN in patients with uncontrolled BP off medication or on up to two classes, reported identical baseline-adjusted between-group differences in daytime ambulatory systolic BP of 6.3 mmHg (95% CI −9.4 to −3.1 and −9.3 to −3.2, respectively) [14,15]. The individual patient pooled analysis of SOLO/TRIO/RADIANCE II [13], and the meta-analyses of second-generation sham-controlled trials [19,20] consistently identify a 4-6 mmHg ambulatory and 8-10 mmHg office systolic BP effect, independent of resistant phenotype. Sub-analyses of the Global SYMPLICITY Registry show that BP reduction occurs irrespective of the number and class of antihypertensives at baseline, and that more patients reduce than increase their drug burden after RDN [30,31].

These data do not prove that all drug-intolerant patients will respond, but show that RDN produces a modest but reproducible ambulatory systolic BP reduction, broadly comparable to adding one standard antihypertensive drug [16,19,20,24]. Such an effect, however modest, is clinically meaningful: large-scale meta-analyses show that each 5 mmHg reduction in systolic BP lowers the risk of major cardiovascular events by approximately 10% over 4 years, irrespective of baseline BP [32]. When pharmacological escalation is not tolerated, RDN may be the only realistic available add-on therapy.

### 1.3 Response heterogeneity and the non-responder problem

Depending on the responder definition used, 25-40% of treated patients derive no meaningful BP reduction, and the individual response distributions of the RDN and sham arms overlap widely in the sham-controlled trials [12,19,20,33].

No pre-procedural variable reliably identifies responders. Higher baseline BP, elevated 24-h heart rate, a non-dipping nocturnal profile, orthostatic hypertension, preserved arterial compliance and markers of sympathetic activation have each been associated with greater response in post-hoc or observational analyses, but none has shown sufficient discriminative performance at the individual level for clinical use [12,33,34]. Higher baseline BP remains the most consistent correlate of a larger absolute reduction, and it is not specific to RDN.

Drug intolerance should therefore not be understood as a marker of RDN responsiveness. It defines a population with an unmet therapeutic need and no pharmacological escalation pathway, but it does not predict the BP response to denervation. A predictable objection is that emerging pharmacological classes (aprocitentan, aldosterone synthase inhibitors such as baxdrostat, RNA-targeted therapies such as zilebesiran) might fill the gap before RDN is considered. These agents enrich the therapeutic arsenal, but three considerations limit their relevance for the multi-class-intolerant patient. First, they overlap mechanistically with classes that have already proven poorly tolerated in this subgroup (sodium retention, hyperkalemia, orthostatic hypotension). Second, their long-term tolerability profile remains incompletely characterized. Third, their efficacy still depends on continued drug exposure. This argument is weaker for long-acting agents such as zilebesiran, given every six months, where the main limitation is tolerability. RDN complements these agents rather than competing with them, in a population where drug-based options reach their limit.

### 1.4 Magnitude of expected benefit and procedural risk

Reported BP reductions after RDN require careful interpretation along two axes. First, the fall from baseline observed in treated patients markedly exceeds the sham-corrected effect, because the former also incorporates placebo response, regression to the mean and Hawthorne effects. In SPYRAL HTN-OFF MED Pivotal, office SBP fell by 9.2 mmHg in the RDN arm but also by 2.5 mmHg in the sham arm, for a baseline-adjusted between-group difference of 6.5 mmHg [16]. In RADIANCE II, the reduction in daytime ambulatory SBP was 7.9 mmHg in the RDN arm versus 1.8 mmHg in the sham arm, a baseline-adjusted between-group difference of 6.3 mmHg [14].

Second, office and ambulatory measurements are not interchangeable. Office readings consistently yield larger reductions than 24-h ambulatory monitoring, being more susceptible to white-coat variability; ambulatory BP, the primary endpoint of all second-generation trials, provides the more conservative and reproducible estimate of true effect. Across sham-controlled trials, the device-attributable ambulatory systolic effect falls between approximately 2 and 7 mmHg, with pooled estimates of 4-6 mmHg, against an 8-10 mmHg office effect [13,19,20]. The projected cardiovascular benefit of this BP reduction is modest in absolute terms. The BPLTTC individual participant-level meta-analysis reports an approximately 10% relative reduction in major cardiovascular events per 5 mmHg reduction in systolic BP [32,35]. Applied to a sustained sham-corrected ambulatory reduction of 4-6 mmHg, this corresponds to a relative reduction of 8-12%. In a patient at high cardiovascular risk, defined as SCORE2 ≥10% at 10 years, an 8-12% relative reduction of a 10% baseline absolute risk yields an absolute risk reduction of 0.8-1.2% and a number needed to treat of the order of 83-125 over ten years. This estimate rests on assumptions that no RDN trial has tested: that the BP effect is sustained over a ten-year horizon, that the BPLTTC relationship, derived over a median follow-up of approximately four years, can be transposed to ten years, that it holds below its 5 mmHg increment, and that a millimetre of mercury lowered by denervation confers the same prognostic benefit as one lowered pharmacologically. No RDN trial has been powered for hard cardiovascular outcomes, and BP reduction remains a validated surrogate rather than a demonstrated outcome benefit.

In a prospective propensity-matched analysis, Zeijen et al. compared 53 RDN-treated patients with 238 hypertensive controls from the population-based Rotterdam Study and found no significant between-group difference in the five-year composite of myocardial infarction, coronary revascularization, stroke, renal failure and mortality (hazard ratio 0.93, 95% CI 0.36–2.38; p = 0.87) [36]. With 51 events in total and only 7 in the RDN arm, the study was powered for blood pressure change, not for clinical events.

RDN safety concerns in this lightly-treated population mirror those in resistant cohorts. The absolute procedural risk nonetheless remains low. Access-site complications occur in ∼1% of cases [37]; in TARGET BP I, a single accessory renal artery dissection was reported among 148 patients treated with alcohol-mediated RDN (0.7%) [28]. Meta-analyses show no significant decline in eGFR after RDN [38], and post-procedural renal artery stenosis after radiofrequency RDN occurs at an annual rate of ∼0.2%, similar to the background rate in hypertension [39].

### 1.5 Guideline positions beyond resistant hypertension

The paradigm that RDN should be offered only for resistant hypertension is increasingly at odds with the recommendations of every major scientific society published since 2023 (Table 2). The 2024 ESC guidelines assign a Class IIb, Level A recommendation to RDN in patients with both increased cardiovascular risk and uncontrolled hypertension on fewer than three drugs, after shared decision-making and multidisciplinary discussion in an experienced center [9]. The same guideline issues a Class III, Level C recommendation against RDN when eGFR is below 40 mL/min/1.73 m^2^ or when a secondary cause is present. The 2025 AHA/ACC guideline assigns a Class IIb, Level B-R recommendation to RDN in carefully selected patients with office systolic BP 140–180 mmHg, diastolic BP ≥ 90 mmHg and eGFR ≥40 mL/min/1.73 m^2^ who have resistant hypertension despite optimal treatment, or intolerable side effects to additional antihypertensive drug therapy [10]. Its patient-selection table explicitly lists patients “unable to take antihypertensive medications at the optimal dosages or additional medications” among appropriate candidates. Multidisciplinary evaluation, screening for secondary causes and shared decision-making each carry a Class 1 recommendation [10]. The 2024 JSH/CVIT/JCS Joint Consensus went further by introducing the category of “conditioned uncontrolled hypertension”, defined as medicated, but uncontrolled BP with either inability to up-titrate due to side effects, comorbidities or reduced quality of life, explicitly including intolerance to CCBs, ACEi/ARBs, thiazide-like diuretics, MRAs or β/α-blockers or cardiovascular comorbidities associated with sympathetic activation [40]. This category was not retained in the 2026 Japanese appropriate-use guideline, which restricts the approved indication to true resistant hypertension defined by three or more antihypertensive classes including a diuretic, with eGFR ≥40 mL/min/1.73 m^2^, out-of-office confirmation of uncontrolled BP and eligibility determined by a certified multidisciplinary team [41]. The 2021 ESH position paper, the 2023 ESH guidelines [8,42], the 2024 SFHTA expert consensus [43], the 2024 Austrian Society of Hypertension recommendations [44] and the SCAI 2023 position statement [45], all recognize drug intolerance as an indication to consider RDN within a multidisciplinary process.

**Table 2:** Current positions of major hypertension guidelines and consensus statements on RDN in non-resistant uncontrolled hypertension and/or drug intolerance.

Society /Year	Recommendation strength	Position on intolerance /non-resistant uncontrolled HTN
NICE 2023 (UK) [46]	Restrictive; no class assigned	RDN restricted to resistant hypertension defined as BP uncontrolled on ≥3 antihypertensive classes. Intolerance not addressed.
ESC/EAPCI 2023 consensus [23]	Clinical consensus statement (no class or level assigned)	Patients non-adherent or intolerant to multiple antihypertensive drugs, particularly first-line agents and spironolactone, may be candidates, and “may therefore be on fewer than 3 drugs at the time of their selection for RDN due to their prior drug intolerance”. Patients with isolated systolic hypertension were excluded from the sham-controlled trials.
ESH 2023 [8]	Class II /Level B for the procedure; Class I /Level C for patient selection by shared decision-making and for performance in experienced centers	RDN can be considered in uncontrolled BP with eGFR >40 mL/min/1.73 m^2^ if drug treatment elicits serious side effects and poor quality of life.
SCAI 2023 [45]	Position statement	Recommends considering RDN not only in resistant hypertension but also in patients unable or unwilling to tolerate antihypertensive medication, in experienced centers.
JSH/CVIT/JCS 2024 consensus [40] and 2026 appropriate-use guideline [41]	Expert consensus, no class assigned to the procedure (2024). JSH 2025: recommendation strength 2, evidence level B, consensus rate 100% (2026 document)	The 2024 consensus introduced “conditioned” uncontrolled hypertension, defined as inability to up-titrate because of side effects, comorbidities or reduced quality of life, explicitly including intolerance to CCBs, ACEi/ARBs, thiazide-like diuretics, MRAs and β/α-blockers. This category was not retained in the 2026 approved indication, which is restricted to true resistant hypertension (≥3 classes including a diuretic, eGFR ≥40 mL/min/1.73 m^2^, office BP ≥ 140/90 mmHg confirmed by out-of-office measurement).
ESC 2024 [9]	IIb/A for uncontrolled BP on fewer than three drugs; IIb/B for resistant hypertension; III/C when eGFR <40 mL/min/1.73 m^2^ or a secondary cause is present	RDN may be considered in patients with increased cardiovascular risk AND uncontrolled BP on fewer than three drugs, after shared risk–benefit discussion and multidisciplinary assessment in a medium-to-high volume center. The supporting text extends this to patients intolerant to multiple BP-lowering medications, particularly first-line agents, with high predicted cardiovascular risk.
Austrian Society of Hypertension 2024 [44]	Position paper (adopts ESH 2023 classes)	RDN may be considered in uncontrolled hypertension with eGFR >40 mL/min/1.73 m^2^ when drug treatment causes serious side effects and poor quality of life, and in grade 1 hypertension specifically in the presence of intolerance to several antihypertensive classes.
SFHTA (France) 2024 [43]	Expert consensus; no own class, explicitly adopts ESH 2023 Class II B	Uncontrolled hypertension with severe drug intolerance impairing quality of life listed as an indication, on a case-by-case basis after multidisciplinary discussion in an ESH Center of Excellence or affiliated BP Clinic.
AHA/ACC 2025 [10]	Class IIb/Level B-R (procedure). Class 1 applies to multidisciplinary evaluation, secondary-cause work-up and shared decision-making, not to the procedure	The recommendation itself covers patients with resistant hypertension despite optimal treatment “or intolerable side effects to additional antihypertensive drug therapy”. Candidates explicitly include patients “unable to take antihypertensive medications at the optimal dosages or additional medications”. Requires office SBP 140–180 mmHg AND DBP ≥90 mmHg, and eGFR ≥40 mL/min/1.73 m^2^.

ACEi: angiotensin-converting enzyme inhibitor; ARB: angiotensin receptor blocker; BP: blood pressure; CCB: calcium channel blocker; DBP: diastolic BP; eGFR: estimated glomerular filtration rate; HTN: hypertension; MRA: mineralocorticoid receptor antagonist; RDN: renal denervation; SBP: systolic BP.

Taken together, contemporary guidelines have moved from a strictly resistant-hypertension-only paradigm toward a phenotype-based framework that includes uncontrolled BP on fewer drugs, intolerance, high cardiovascular risk and patient preference.

### 1.6 Persistent barriers and remaining uncertainties

A common objection, even when efficacy and safety are accepted, is the upfront cost of the procedure relative to drugs. The published health-economic evidence does not support this concern. Three contemporary country-specific analyses, all using Markov models calibrated to the SPYRAL HTN-ON MED and HTN-OFF MED trial data, converge on the same conclusion. In the UK analysis, radiofrequency RDN had an incremental cost-effectiveness ratio (ICER) of £13,482 per quality-adjusted life-year (QALY) gained, with a 100% probability of being cost-effective below the £20,000/QALY NICE threshold [47]. In the corresponding US analysis, the ICER was $32,732/QALY, well below the conventional $50,000/QALY high-value threshold [48]. In Japan, the cost-effectiveness analysis yielded an ICER of ¥2,565,236/QALY (≈€15,500/QALY) against a willingness-to-pay threshold of ¥5,000,000/QALY; sensitivity analyses incorporating nighttime BP benefit shifted the ICER further into favorable territory [49]. A recent systematic review and meta-analysis covering 19 cost-effectiveness studies across 16 countries reported every single analysis to favor RDN, with ICERs consistently well below national willingness-to-pay thresholds [50].

These analyses were performed primarily in resistant or broadly uncontrolled hypertension. There are three reasons to expect the economic case to be even stronger in the multi-class-intolerant patient. First, this group is on average younger than the resistant cohort and therefore accumulates more discounted QALYs over the post-procedure horizon. Second, drug costs avoided are real: in the intolerant patient, pharmacological alternatives will involve later-line, more expensive agents. Third, intolerance is itself associated with non-adherence, repeated consultations and emergency department visits for hypertensive crises [4,25,51]. The cost-effectiveness argument therefore extends, rather than weakens, when the indication is broadened to the intolerant patient with high cardiovascular risk. Despite these favorable cost-effectiveness estimates, the 2025 CMS National Coverage Determination (CAG-00470N) makes reimbursement conditional on enrolment in an evidence-generation programme, reflecting the absence of hard-outcome data [52].

If guidelines and economics align in favor of broader assessment, why is uptake still slow? Two recent surveys provide an answer. In a 2025 cross-sectional survey of the Argentine Society of Hypertension (n = 206 cardiologists and internists), 83% of physicians were familiar with RDN, but only 60% considered it safe and 33% remained uncertain [53]. In the 2024 Italian SIIA nationwide survey of 82 hypertension centers, the main barriers were organizational rather than evidentiary: absence of a multidisciplinary team, lack of referral pathways and limited operator availability [54]. This implementation gap is reinforced by a patient-physician perception mismatch: survey data show that patient willingness to consider RDN exceeds physician estimation, particularly when drug-related side effects are present [[55], [56], [57]] (Supplementary Fig. 1).

The absence of dedicated cardiovascular outcome trials in non-resistant drug-intolerant patients is real, but this argument applies broadly across hypertension therapeutics. BP lowering remains the accepted surrogate for antihypertensive efficacy, supported by large meta-analyses showing consistent risk reduction per mmHg of systolic BP lowering [32,35].

### 1.7 True intolerance versus pseudo-intolerance

Unlike resistant hypertension, drug intolerance lacks an objective definition. A substantial proportion of reported intolerance is not pharmacologically attributable: n-of-1 and rechallenge studies show that many drug-attributed symptoms also occur on placebo [58]. Both apparent intolerance and apparent resistance frequently reflect undisclosed non-adherence, detected by biochemical testing in a pooled 37% of apparent treatment-resistant patients, rising to 46% with direct urine or serum LC-MS/MS methods [59]. The 2025 CMS Determination requires before referral the exclusion of pseudo-resistance: out-of-office confirmation of uncontrolled BP, a stable period of maximally tolerated guideline-directed therapy with documented adherence assessment and screening for secondary causes [52].

### 1.8 Toward a pragmatic implementation framework

We propose that RDN should be offered to patients who meet all of the following criteria (Fig. 2).

• Exclusion of non-adherence by direct measurement of antihypertensive drugs or their metabolites in urine or serum.
• Documented failure of a low-dose single-pill combination, prescribed for four to eight weeks, with assessment of both BP response and tolerability.
• Documented intolerance to at least two first-line antihypertensive classes (RAS blocker, CCB, thiazide/thiazide-like diuretic), with chart-documented adverse events that prevented up-titration or required discontinuation.
• Uncontrolled BP confirmed by repeated out-of-office BP assessment, preferably ABPM when available (24-h ≥130/80 mmHg, daytime ≥135/85 mmHg, or nighttime ≥120/70 mmHg), on the maximum pharmacological regimen tolerated.
• Increased cardiovascular risk defined by any of: established atherosclerotic cardiovascular disease, type 2 diabetes with target-organ damage, hypertension-mediated organ damage, CKD stage 3 with eGFR ≥40 mL/min/1.73 m^2^, SCORE2 ≥10% over 10 years.
• Secondary hypertension excluded with structured screening for primary aldosteronism, renal artery stenosis, obstructive sleep apnea and drug-induced hypertension.
• Eligible renal anatomy on CT or MR angiography with adequate renal artery diameter, no severe stenosis, no prior stenting and no fibromuscular dysplasia.

**Fig. 2:**
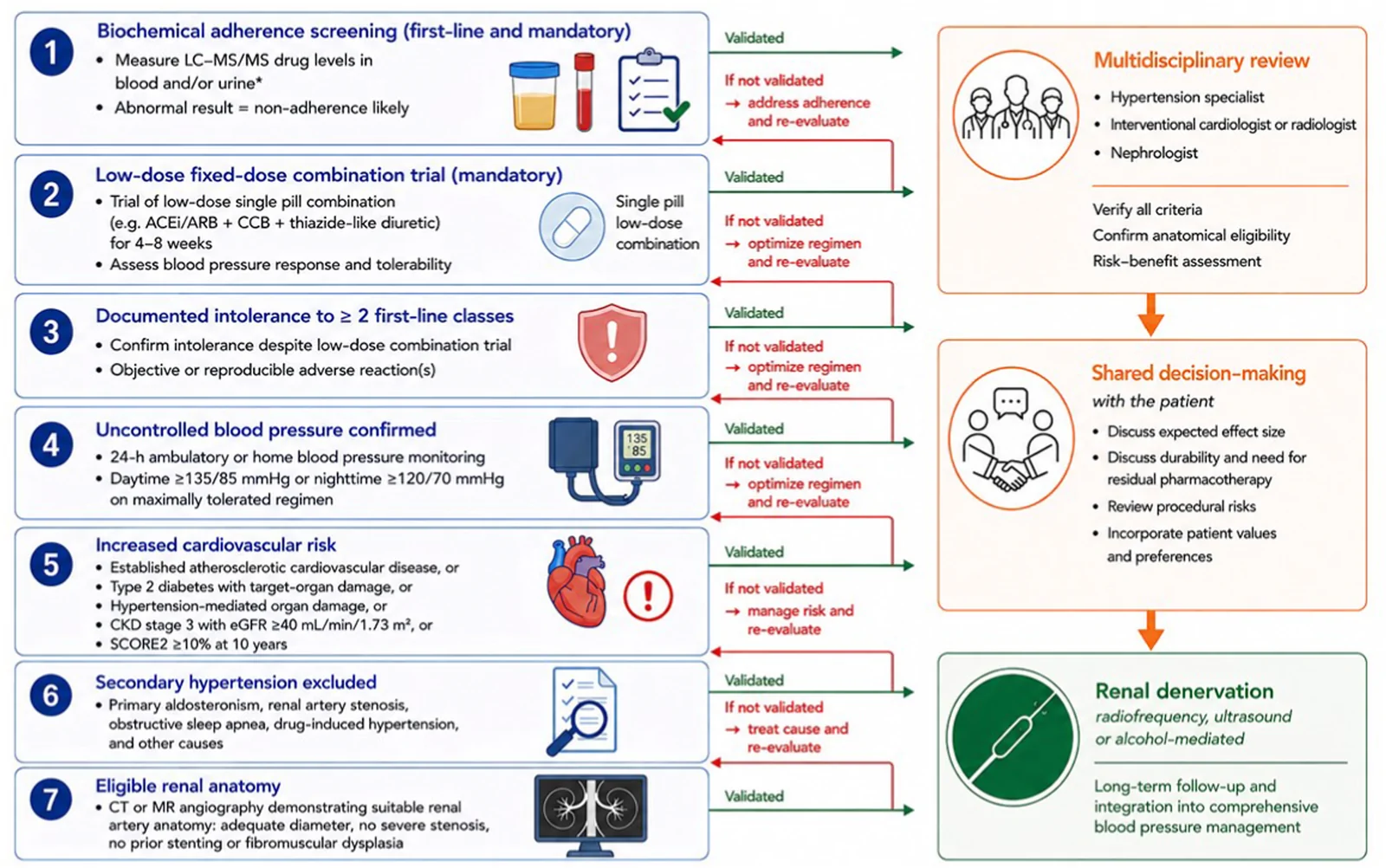
Proposed referral and shared decision-making algorithm for patients with uncontrolled hypertension and multiple drug intolerances. *Biochemical adherence testing is not standardized across centers; assay availability and interpretation thresholds vary. ACEi: angiotensin-converting enzyme inhibitor; ARB: angiotensin receptor blocker; CCB: calcium channel blocker; CT: computed tomography; eGFR: estimated glomerular filtration rate; LC-MS/MS: liquid chromatography–tandem mass spectrometry; MR: magnetic resonance.

Patients meeting all criteria should undergo multidisciplinary review in an ESH Center of Excellence or equivalent expert center, followed by shared decision-making with explicit discussion of expected effect size, durability, residual need for pharmacotherapy when tolerated, the possibility of non-response and procedural risk [43,53,54].

## 2 Conclusion

Restricting renal denervation to resistant hypertension preserves a definition that no longer fully reflects the contemporary hypertension phenotypes, the available trial evidence, or the current guideline positions. We propose that patients with uncontrolled BP and documented drug intolerance, in whom non-adherence has been excluded biochemically, should become candidates for structured assessment to RDN. The expected benefit is modest, and hard-outcome data are lacking; these limitations should be presented explicitly during shared decision-making. Withholding RDN until a dedicated randomized trial in multi-class-intolerant patients is completed would nonetheless leave this subgroup without a non-pharmacological adjunctive option for the next years. The remaining question is not whether such patients may benefit from RDN, but how to operationalize their identification, referral and shared decision-making within multidisciplinary care. This proposal does not resolve a more fundamental limitation. Hypertension is a heterogeneous vascular syndrome, comparable in phenotypic complexity to heart failure with preserved ejection fraction, in which the contributions of sympathetic overactivity, renin-angiotensin-aldosterone activation, arterial stiffening and sodium handling vary between individuals; RDN targets a single axis, which likely explains the wide response variability observed when patients are selected on BP level rather than mechanism. Progress will require prospective validation of markers of sympathetically-driven hypertension capable of identifying likely responders before the procedure, such as muscle sympathetic nerve activity, noradrenaline spillover or imaging of renal innervation. Integrating these measures into RDN registries and trials would support a shift from a BP-threshold indication toward a mechanism-based one.

## CRediT authorship contribution statement

**Elena-Mihaela Cordeanu:** Writing – review & editing, Writing – original draft, Visualization, Validation, Project administration, Methodology, Investigation, Conceptualization. **Emma Morisot:** Writing – original draft, Visualization, Validation, Investigation. **Corina Mirea:** Writing – original draft, Visualization, Validation, Methodology, Conceptualization. **Eric Prinz:** Writing – original draft, Visualization, Investigation, Conceptualization. **Francois Bronner:** Writing – original draft, Supervision, Methodology. **Dominique Stephan:** Writing – review & editing, Writing – original draft, Visualization, Project administration, Conceptualization.

## Data availability

Not applicable. This is a controversies article based on published literature.

## Declaration of generative AI and AI-assisted technologies in the writing process

During the preparation of this work the authors used Claude AI and ChatGPT in order to improve language and readability of the manuscript and for figure creation upon prompts designed and written by the authors. After using this tool, the authors reviewed and edited the content as needed and take full responsibility for the content of the publication.

## Funding

This research did not receive any specific grant from funding agencies in the public, commercial, or not-for-profit sectors.

## Declaration of competing interest

The authors declare that the research was conducted in the absence of any commercial or financial relationships that could be construed as a potential conflict of interest.

## Figures and Tables

**Figure:**
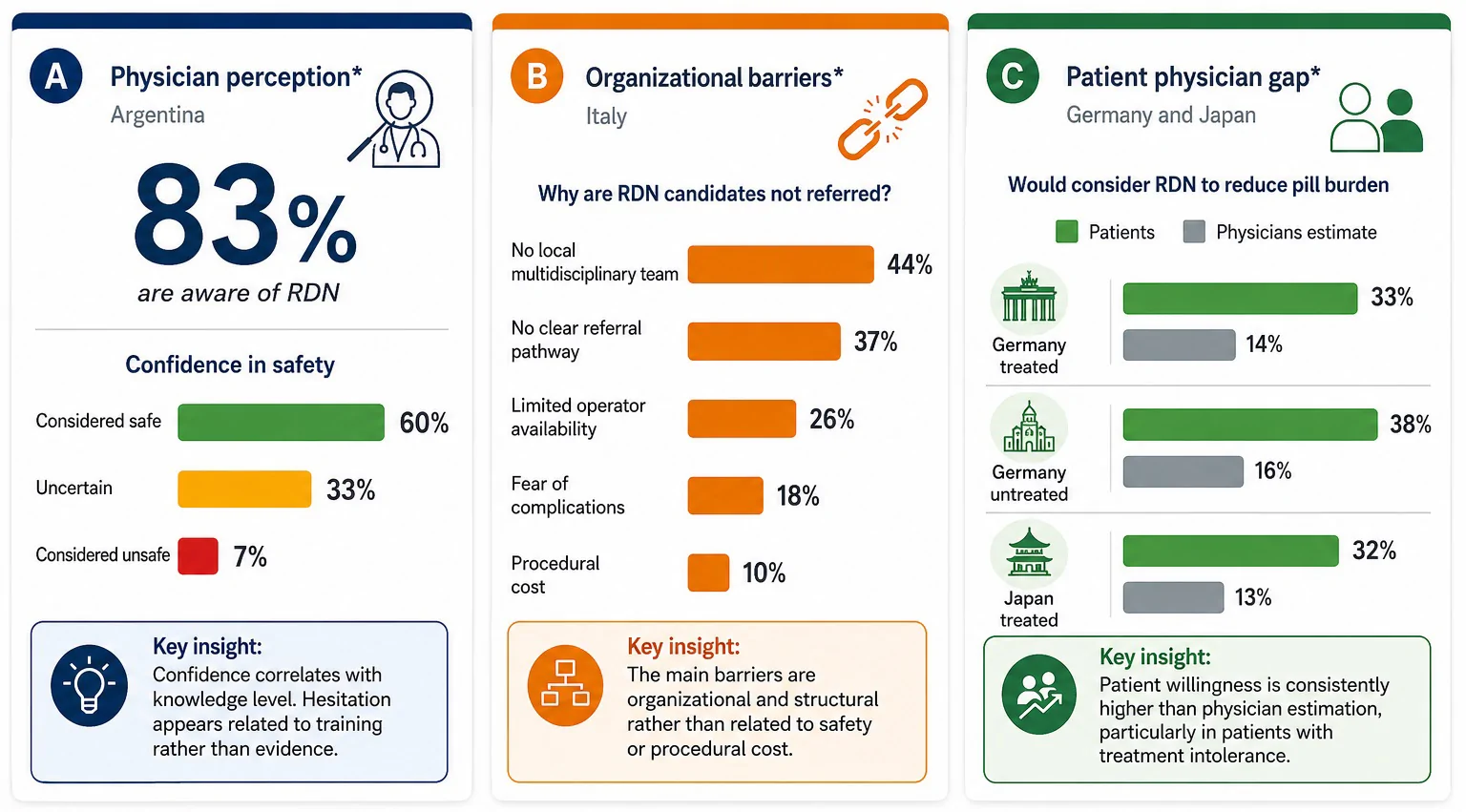
Supplementary Figure 1. The implementation gap: physician awareness, organizational barriers and the patient–physician perspective mismatch. Panel A, Argentine Hypertension Society survey; Panel B, Italian SIIA nationwide survey; Panel C, physician-patient perception gap. RDN: renal denervation.
